# Influence of the Addition of Trace Amounts of Vinylpyrrolidone–Vinyl Acetate Copolymer (PVPVA) on the Crystallization of Celecoxib Glass

**DOI:** 10.1021/acs.molpharmaceut.5c00934

**Published:** 2025-12-15

**Authors:** Xue Han, Kaoru Ohyama, Kohsaku Kawakami

**Affiliations:** † Research Center for Macromolecules and Biomaterials, 52747 National Institute for Materials Science, 1-1 Namiki, Tsukuba, Ibaraki 305-0044, Japan; ‡ Graduate School of Science and Technology, University of Tsukuba, 1-1-1 Tennodai, Tsukuba, Ibaraki 305-8577, Japan

**Keywords:** glass, polymeric excipients, PVPVA, crystallization, broadband dielectric spectroscopy, differential scanning calorimetry

## Abstract

Although polymers can prevent the crystallization of glassy drugs in amorphous solid dispersions, their stabilization mechanism requires further clarification for an efficient formulation design. This study examined the impact of adding trace amounts (2 or 5 w/w %) of vinylpyrrolidone–vinyl acetate copolymer (PVPVA) on the physical stability of celecoxib (CEL) glass using differential scanning calorimetry and broadband dielectric spectroscopy. Long-term isothermal crystallization studies from 35 to 60 °C revealed that CEL glass was significantly stabilized by the addition of trace amounts of PVPVA. Its stabilization was attributed to the effect of PVPVA on the nucleation process rather than on crystal growth. The addition of PVPVA slowed down the α-relaxation of CEL, whereas it accelerated Johari–Goldstein relaxation. Moreover, the addition of PVPVA effectively slowed down γ*-* and δ-relaxations. Of these, suppression of γ-relaxation mobility had the most important effect, as it is related to the formation of hydrogen bonding between CEL and PVPVA molecules to inhibit nucleation. Moreover, the change in molecular cooperativity of the CEL glass upon adding PVPVA contributed to the inhibition of nuclei formation due to the decreased nucleation temperature. This study provides detailed insights into the physical stabilization mechanisms of glass using polymeric excipients.

## Graphical abstract

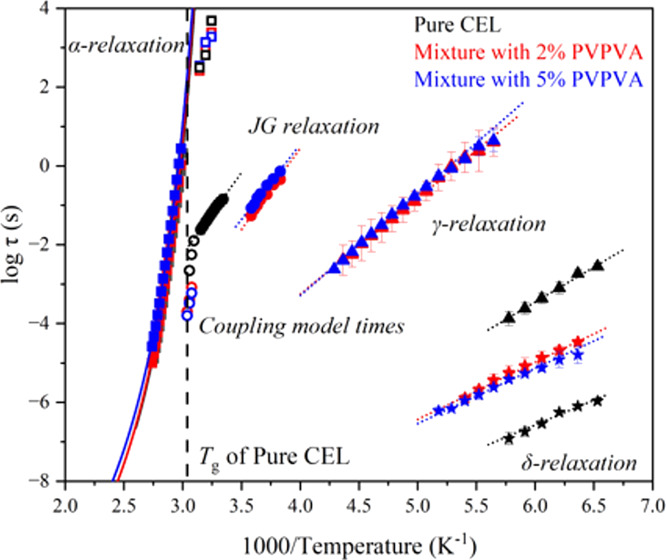

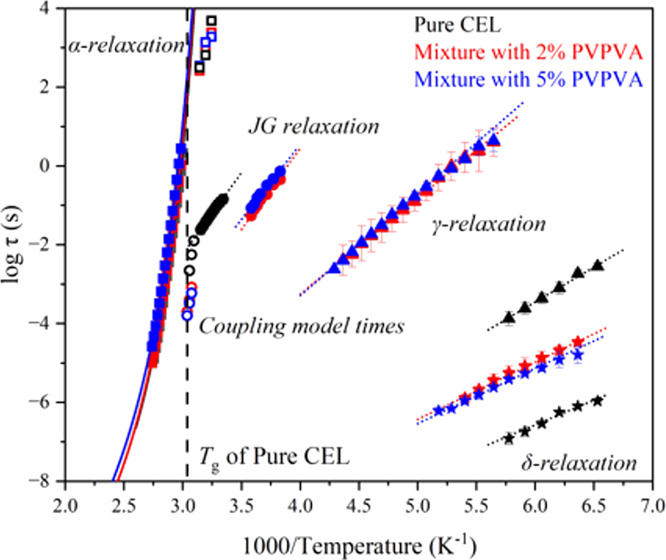

## 1 Introduction

Poorly soluble drugs may offer limited oral bioavailability when administered as crystalline formulations.^1,2^ To address this issue, the use of the glassy state, which exhibits a higher Gibbs energy and increased solubility compared to its crystalline counterparts, is a promising strategy.^3^ However, the crystallization of glassy drugs during storage or after administration diminishes their solubility advantage.^4^ To maintain desired drug properties, an effective method to improve physical stability involves dispersing active pharmaceutical ingredients (APIs) within the polymer matrix to construct amorphous solid dispersions (ASDs).^5^ Polymers highly compatible with APIs and highly soluble to aqueous media serve as inhibitors for crystallization in the solid state and when in contact with aqueous media.^6^ For example, nimesulide glass containing 5% inulin, Soluplus, or polyvinylpyrrolidone (PVP) required 1.8, 6.7, or 8.8 h, respectively, to reach 50% crystallinity, although pure nimesulide only required 33 min at 55 °C.^7^ The addition of 5–10% PVP prolonged the time required to reach 90% crystallinity for nifedipine and phenobarbital glasses by 100–1000 times.^8^ During supersaturation, the felodipine–hydroxypropyl methylcellulose (HPMC) ASD exhibited crystallization inhibition capacity, with a lower recrystallization rate of 0.1 μg/mL/min compared to that of 0.13 μg/mL/min observed for samples without the polymer.^9^

Certain mechanisms have been suggested to explain the enhancement of physical stability. For example, antiplasticization occurs when the glass is dispersed into a polymer matrix with a higher glass transition temperature (*T*_g_). If the mixing is favorable, the APIs will be evenly dispersed in the system to lower the Gibbs energy of the system.^10^ Molecular interactions such as hydrogen bonds, electrostatic forces, and ionic interactions also play crucial roles in stabilizing APIs. These interactions limit API recrystallization by hindering crucial intermolecular interactions required for nucleation and crystal growth initiation.^11^ In addition, polymers decrease the molecular mobility of APIs by increasing viscosity, thereby decreasing the rates of nucleation and crystal growth of APIs.^12^

Extensive researches have been conducted on the solubility of APIs in polymer matrix to avoid recrystallization over extended periods of storage^13^ for designing homogeneous ASDs. For example, a concentration of 50% polyvinylpyrrolidone–vinyl acetate (PVPVA) was required to solubilize flutamide.^14^ At this concentration, no crystallization tendency was observed for more than 270 days at room temperature. The solubilities of flutamide in poly­(vinyl acetate) and PVP to enable complete stabilization were 35% and 71%, respectively.^15^ Those of aripiprazole in PVPVA and Soluplus were as low as 30% and 15%, respectively. No crystallization was observed after 220 days of storage at 25 °C.^16^ However, low solubility inevitably results in an increase in the required dosage.

Several investigations indicate that even the addition of a small amount of polymer shows a significant stabilization effect on amorphous APIs. The crystallization rate of indomethacin was reduced in the presence of 1% PVP, which means that 31 indomethacin molecules were stabilized by one vinylpyrrolidone unit. This suggests that stabilization arises not from direct interaction between two molecules but presumably because PVP inhibited crystal growth from the surface to the inner glass.^17^ Moreover, indomethacin could maintain a glass state at 30 °C for 14 months in the presence of 5% PVP, which was associated with PVP–indomethacin molecular interactions.^18^ The growth rate of nifedipine glass was significantly suppressed in the presence of 1% PVP.^19^ The nucleation rate of fluconazole was inhibited by 10% hydroxypropyl methylcellulose acetate succinate but increased in the presence of poly­(ethylene oxide).^20^ Discrimination of surface and bulk growth may provide further insights. Bulk crystal growth of nifedipine slowed by 10 times in the presence of PVP, while surface crystal growth slowed by only 2 times.^21^ In this study, we provide a more detailed investigation on both nucleation and crystal growth processes in the presence of trace amounts of polymer, which should provide a clearer strategy for stabilization of ASDs using a small amount of polymeric excipients.

## 2 Experimental Section

### 2.1 Materials

CEL (Form III) was purchased from Tokyo Chemical Industry and used without further purification. *T*_g_ of CEL glass is ca. 58 °C.^22,23^ PVPVA (Kollidon VA 64) was obtained from the BASF Corporation (Ludwigshafen am Rhein, Germany). The molecular weights for CEL and PVPVA are 381 and 45000–70000 g/mol, respectively. Their chemical structures are listed in Figure 1.

**Figure 1:**
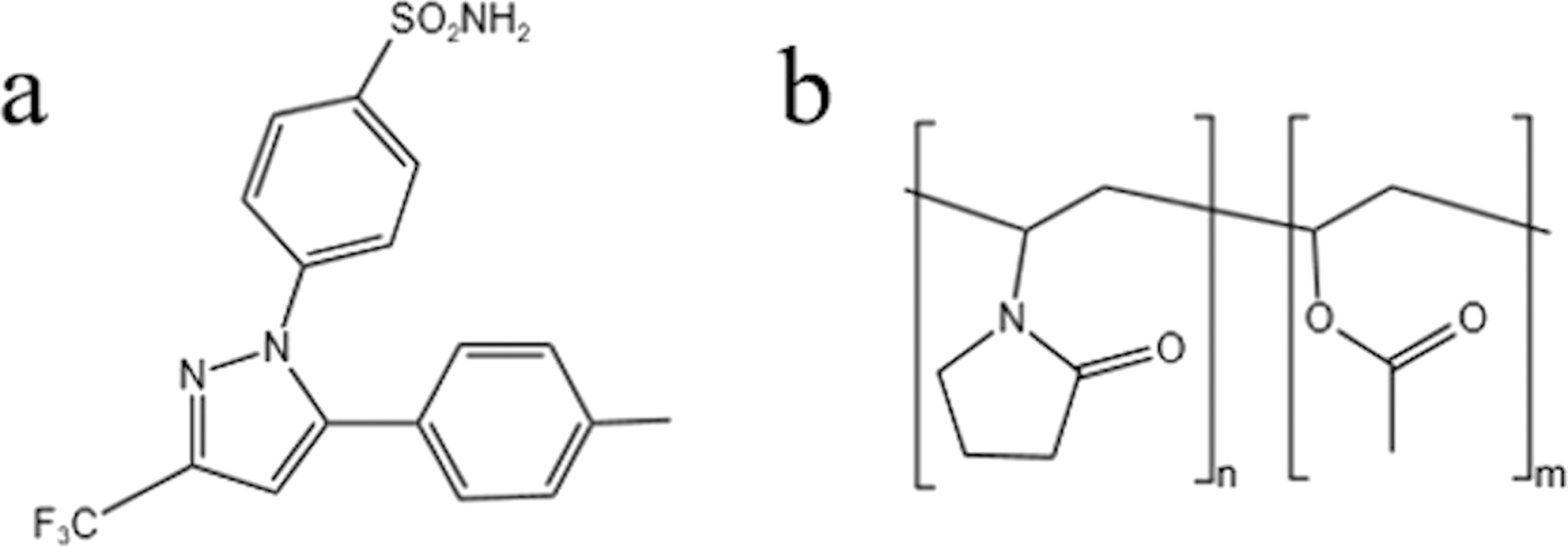
Chemical structures of (a) CEL and (b) PVPVA.

### 2.2 Preparation of the Glass Samples

Binary mixtures were prepared by carefully mixing CEL and PVPVA in various ratios using a mortar and pestle for 20 min. The samples were melted at 170 °C on a hot plate, followed by cooling in an ambient atmosphere at 25 °C to obtain a glass state. To avoid the sorption of moisture prior to long-term annealing tests, the samples were placed in a sealed box with silica gel. The mixing ratios are expressed as the proportion of PVPVA used in this study. For instance, a mixture of CEL and PVPVA at a weight ratio of 98:2 is termed a mixture with 2% PVPVA.

### 2.3 Differential Scanning Calorimetry (DSC)

DSC (Q2000, TA Instruments, New Castle, DE, USA) was employed for thermal analysis. The instrument was calibrated using indium and sapphire, and dry nitrogen was used as the inert gas at a flow rate of 50 mL/min. Approximately 5 mg of the sample was sealed in an aluminum pan and heated to 180 °C at a heating rate of 10 °C/min for melting. The sample was then cooled to −20 °C at a rate of 10 °C/min to induce nucleation. The glass transition and cold crystallization of CEL were observed during the second heating at a rate of 10 °C/min.^22,23^ All experiments were repeated three times. All enthalpy values are expressed per gram of the total mixture mass.

The melting peaks of the CEL crystal frequently included those for Forms I and III, which were overlapped. The overlapping melting peaks indicate either independent melting of the two forms or melt-crystallization. For CEL, the intensity ratio of the two melting peaks depended on the annealing conditions of the glassy state,^22^ suggesting that the melting of the two forms independently occurred. Thus, deconvolution was performed using two Lorenz peaks.^24^

### 2.4 Size of the Cooperatively Rearranging Region (CRR)

The size of the CRR was determined by using DSC in the temperature-modulated mode. The samples were sealed in Tzero pans and heated to 180 °C at a rate of 10 °C/min for melting, followed by cooling at 10 °C/min to −50 °C. The samples were then heated at 2 °C/min under a modulated mode with an amplitude and period of 0.5 °C and 60 s, respectively. Three independent samples were analyzed for each composition. The CRR size (*L*) was determined using the following equation:^25,26^

1 $$L={\left\{\frac{3k{T_{g}}^{2}}{4\pi \rho \Delta d_{T_{g}}^{2}}\left(\frac{1}{C_{pg}}-\frac{1}{C_{pl}}\right)\right\}}^{1/3}$$

where ρ and $\Delta d_{T_{g}}$ are the density and half of the glass transition width, respectively; *T*_g_ is the onset glass transition temperature; *k* is Boltzmann’s constant; and *C*_pg_ and *C*_pl_ are the specific heat capacities of the glass and supercooled liquid, respectively. According to a previous study,^27^ *C*_pg_ and *C*_pl_ were 1.62 and 2.07 J/(g°C), respectively. The glass transition width was the difference between the onset and end points of *T*_g_. The density of the CEL glass was determined to be 1.41 g/cm^3^ in our previous study using a helium pycnometer.^22^

### 2.5 Determination of α-Relaxation Time

A relaxation study was performed on DSC to determine the α-relaxation time. After melting at 180 °C, the samples were cooled to an annealing temperature (*T*_a_) at a rate of 20 °C/min. After the annealing for a predetermined period, the samples were subjected to the measurement using the modulated mode at 2 °C/min with a period and amplitude of 60 s and 0.5 °C, respectively.

The recovery enthalpy value of the sample was calculated by the subtraction from enthalpy of the nonreversing heat flow of the annealed sample to that of the nonannealed sample. The relaxation function, Φ, was determined using the following equation:^28^

2 Φ=1−ΔHtΔH∞

where Δ*H*_t_ is the enthalpy recovery measured under the given conditions, and Δ*H*_∞_ is the maximum enthalpy recovery calculated using the following equation:

3 $$\Delta H_{\infty }=\Delta C_{p}\left(T_{g}-T_{a}\right)$$

The mean relaxation time was calculated by fitting the data to the Kohlrausch–Williams–Watts (KWW) equation:

4 Φ=exp{−(tτα)βKWW}

where τ_α_ is the α-relaxation time, and β_KWW_ is used to evaluate the distribution of relaxation time.

### 2.6 Long-Term Physical Stability

Samples in DSC pans were stored in temperature-controlled ovens for predetermined periods for evaluating the long-term physical stability. The melted samples were initially stored with silica gel in a freezer at −20 °C for 16 h for inducing nucleation. Then, the samples were transferred with silica gel into ovens controlled at 35, 40, 45, 50, and 60 °C. The remaining glass fraction of the samples was determined using the change in heat capacity at *T*_g_ (Δ*C*_p_). Three independent samples were analyzed for each annealing condition.

### 2.7 Determination of Nucleation Temperature

The nucleation temperature of the CEL glass in the presence of PVPVA was determined via annealing tests using DSC.^23^ First, the sample was heated at 20 °C/min to 180 °C and held at this temperature for 1 min. The sample was then cooled to the *T*_a_ at a rate of 20 °C/min and maintained for 5 min or 1 h. Subsequently, the sample was heated at 10 °C/min to 180 °C to observe cold crystallization. Ten samples were evaluated for each *T*_a_ to estimate the probability of crystallization, the cold crystallization enthalpy, and the onset temperature (*T*_onset_) of cold crystallization, except for *T*_a_ = 0 °C for which three samples were measured.

### 2.8 Broadband Dielectric Spectroscopy (BDS)

Dielectric permittivity measurements were performed using a Novocontrol Alpha dielectric spectrometer (Montabaur, Germany) in the frequency range from 10^–2^ to 10^7^ Hz under ambient pressure. The glass samples were prepared by melting mixtures on a stainless-steel sample stage using a hot plate heated to 170 °C followed by quenching under ambient temperature to obtain the glass state. Sample thickness was controlled to 0.1 mm by inserting silica spacer fibers between the stainless-steel plates. The prepared samples were transferred to the dielectric spectrometer followed by reheating at 190 °C for 20 min to completely dehydrate the samples. The measurement temperatures were controlled via the Quatro system using nitrogen gas generated from liquid nitrogen and ranged from −120 to 140 °C. The temperature intervals for measurements were every 4 °C below 0 °C and every 2 °C for over 0 °C. Temperature stability was within ±0.5 °C. All experiments were repeated twice.

The relaxation processes were fitted using the Havriliak–Negami (HN) function to obtain the mean relaxation time,^22^ as follows:

5 $$\varepsilon^{*}\left(\omega \right)=\varepsilon^{\prime }\left(\omega \right)-i\varepsilon^{\prime \prime }\left(\omega \right)=\varepsilon_{\infty }+\frac{{\Delta \varepsilon }_{k}}{{\left[1+{\left(i\omega \tau_{\mathrm{HN}}\right)}^{a}\right]}^{b}}$$

where ε*­(ω) is the complex permittivity; ε′(ω) and ε″(ω) are the real and imaginary parts of the complex dielectric permittivity, respectively; ε_∞_ is the high-frequency limit permittivity; *k* denotes either the primary or secondary process; Δε_*k*_ is the relaxation strength; τ_HN_ is the relaxation time; and *a* and *b* are exponents of the relaxation processes. ω is equal to 2π*f*, where *f* denotes the frequency. For the secondary relaxation process, *b* is fixed to unity as a Cole–Cole (CC) function. The relaxation time, τ_α_, was calculated using the following equation:^29^

6 $$\tau_{\alpha }=\tau_{\mathrm{HN}}\times {[\sin\left(\frac{\pi ab}{2+2b}\right)]}^{1/a}{[\sin\left(\frac{\pi a}{2+2b}\right)]}^{-1/a}$$

### 2.9 Fourier Transform Infrared Spectroscopy (FTIR)

FTIR spectra were acquired by using a Nicolet iS20 FTIR spectrometer (Thermo Fisher Scientific, Waltham, MA, USA) with an attenuated total reflection attachment. The spectra were obtained in the range of 400–4000 cm^–1^, averaging data of 64 scans.

### 2.10 X-ray Powder Diffraction (XRPD)

After annealing, the samples were collected and subjected to an XRPD analysis. To confirm the crystal form following cold crystallization in the DSC measurements, the annealed samples were crystallized on a hot plate at 140 °C and subjected to XRPD analysis. The data were obtained on a Rigaku RINT Ultima X-ray Diffraction System (Rigaku Denki, Tokyo, Japan) with Cu K-α radiation. The voltage and current were 40 kV and 40 mA, respectively, and data were acquired at a scan rate of 0.5 °C/min with 0.02° intervals.

### 2.11 Polarized Light Microscopy (PLM)

CEL crystal growth was observed under a hot-stage microscope (Olympus BX-51, Tokyo, Japan) equipped with a U-POT polarizer and a U-ANT analyzer. The sample temperature was controlled by using a PN121-D heat stage (MSA Factory, Tokyo, Japan). CEL and its mixture with 2% PVPVA were melted on a thin glass by using a hot plate heated at 200 °C, followed by cooling to ambient temperature and storage at −20 °C for 12 h in a freezer. The glass samples were stored in an airtight box containing silica gel to protect them from moisture. Subsequently, a cover glass was applied to investigate crystal growth at 120 °C. In the images, 16 radial lines were drawn in equally spaced directions from the center of the crystal, and the linear growth velocity was analyzed.

## 3 Results

### 3.1 Thermal Analysis of CEL Glass and Its Mixtures with PVPVA

Pure CEL and the CEL/PVPVA mixtures were first melted in DSC by heating the sample above the melting temperature of CEL followed by cooling to −20 °C to induce nucleation.^23^ Figure 2a presents the second heating curves. In all samples, a single glass transition was observed, and the *T*_g_ values of the mixtures increased with increasing amounts of PVPVA. The midpoint *T*_g_ values of pure CEL and the 2% and 5% PVPVA mixtures were 55.2, 56.0, and 57.8 °C, respectively. A cold crystallization peak was observed for pure CEL; however, this peak became smaller for the mixture with 2% PVPVA and disappeared for that with 5% PVPVA. This result indicates the strong inhibitory effect on crystallization with only trace amounts of PVPVA. An increase in the onset temperature for crystallization in the presence of PVPVA was also observed in the BDS studies (Figure S1). Figure 2b shows the effect of PVPVA addition on the *T*_g_ and size of the CRR. Adding the polymer decreased the CRR size, which is consistent with our previous observations using ibuprofen glass.^30^ Though the Donth equation has been pointed out that it can have some systematic errors,^31^ the trend of decreasing molecular cooperativity by adding trace amounts of PVPVA was the most likely. Besides, the typical values for organic glasses are ca. 2 nm,^25^ which is consistent with the presented results.

**Figure 2:**
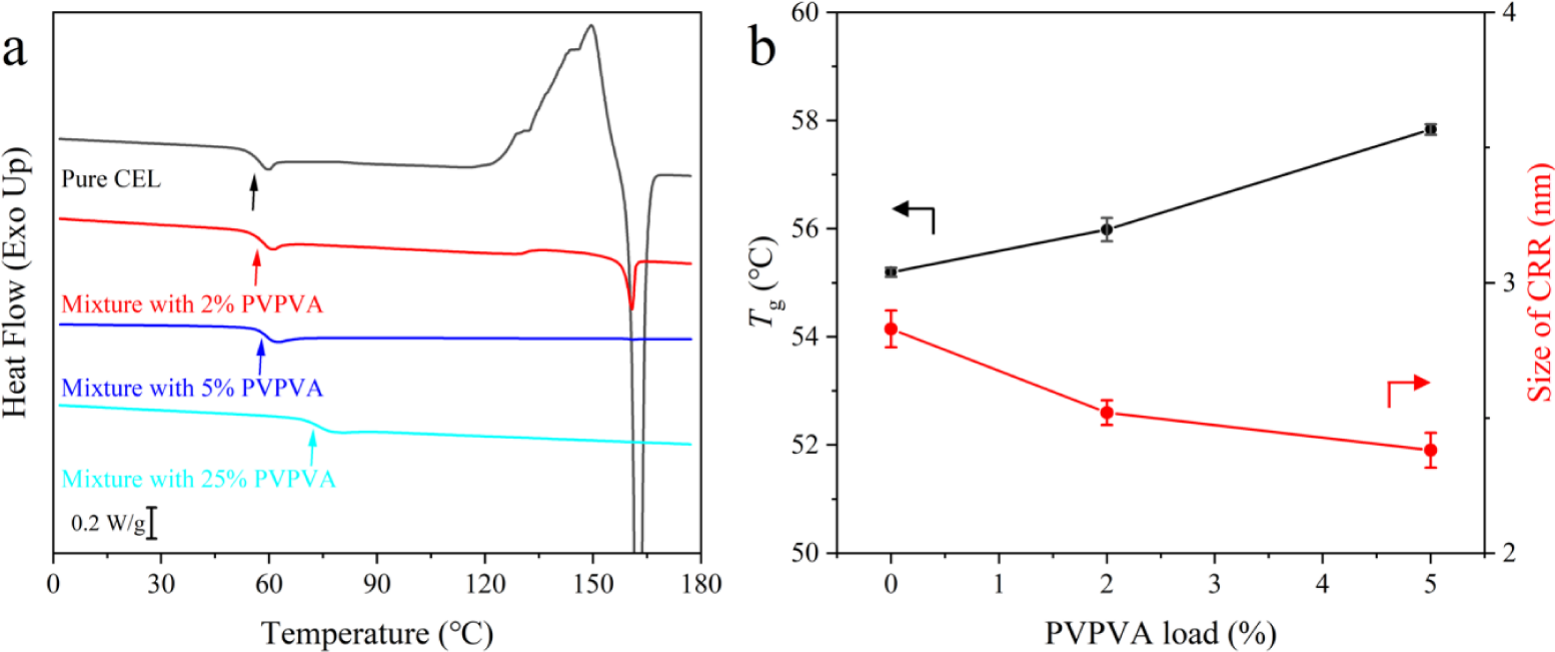
(a) DSC curves of CEL and its mixtures with PVPVA. *T*_g_ is noted using arrows. (b) Effect of adding PVPVA on the *T*_g_ and size of the CRR of CEL glass.

### 3.2 Molecular Mobility of CEL Glass and Its Mixtures with PVPVA in the Supercooled Liquid State

The BDS measurements revealed that all α*-*relaxation peaks in the supercooled liquid state of the samples exhibited identical shapes (Supporting Information) and were fitted well with the HN function. Figure 3a shows the normalized dielectric spectra of CEL glass and its mixtures with PVPVA. The spectra of the mixtures showed a shift to lower frequencies with increasing amounts of PVPVA. The shape of the α*-*relaxation peak was analyzed by fitting using the KWW function to provide information on the distribution of τ_α_. The β_KWW_ values were 0.69, 0.61, and 0.58 for pure CEL glass, a mixture with 2% PVPVA, and a mixture with 5% PVPVA, respectively. Thus, adding PVPVA made the molecular mobility of CEL heterogeneous.

**Figure 3:**
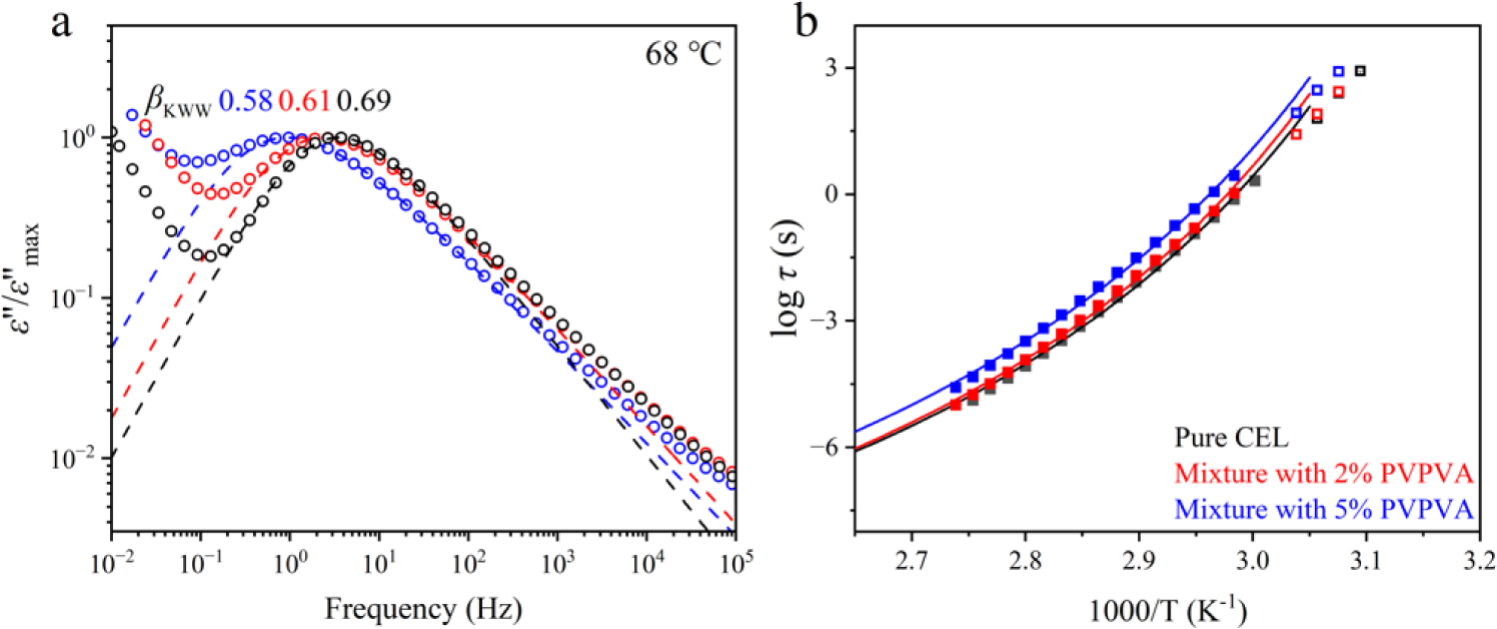
(a) Normalized dielectric loss spectra of pure CEL glass, the mixture with 2% PVPVA, and the mixture with 5% PVPVA at 68 °C. The KWW fits are represented by break lines, and ε″_max_ is the maximum value of the α peak. (b) τ_α_ of each sample at temperature over *Τ*_g_ (closed squares) was fitted using the VFT equation, where τ_α_ = 100 s is defined at *Τ*_g_, and τ_α_ of each sample at temperature below *Τ*_g_ (open squares) was calculated from masterplot (the detail is presented in Supporting Information).

As shown in Figure 3b, the obtained τ_α_ values were fitted using the Vogel–Fulcher–Tammann (VFT) equation, which is expressed as:^26^

7 $$\tau_{\alpha }=\tau_{\infty }\exp\left(\frac{DT_{0}}{T-T_{0}}\right)$$

where τ_∞_ is a pre-exponential factor; *T*_0_ is the Vogel temperature, which appears to be equivalent to the Kauzmann temperature; and *D* is the Angell strength parameter. The data for pure CEL glass were fitted using *T*_0_ = 280 K and *D* = 6.33 under the assumption of logτ_∞_ = −14. Similarly, *T*_0_ values for the mixtures with 2% and 5% PVPVA were 281 and 279 K, with *D* values of 6.30 and 6.83, respectively. If τ_∞_ is treated as a variable, then its value decreases to an unusually small value, ca. −16. Nevertheless, the trend that the addition of PVPVA does not affect the results remains the same.

### 3.3 Molecular Mobility of CEL Glass and Its Mixtures with PVPVA in the Glassy State

Below the *Τ*_g_, α-relaxation was observed using DSC. The consumption process of excess enthalpy over time for both mixtures in the form of Φ is presented in Figure 4a,b, and the obtained KWW parameters by the best fit to eq 4 with those for pure CEL^22^ are listed in Table 1. The relaxation time is shown as a function of *T*_g_/*T*_a_ in Figure 4c. Molecular mobility decreased with increasing PVPVA content. A comparison of the β_KWW_ values indicated that the distribution of τ_α_ for pure CEL rarely depended on the temperature. When PVPVA was present, it widened with increasing temperature. Figure 4c shows the Arrhenius plots of the relaxation times, where $\tau_{\alpha }^{\beta_{\mathrm{KWW}}}$ , calculated by two parameters derived from fitting the KWW equation, was used instead of τ_α_ to diminish the effect of different distributions.^28^ Loading 2% and 5% PVPVA increased the activation energy to 203 and 264 kJ/mol, respectively, compared to 86.7 kJ/mol for pure CEL. These results may be related to decreased molecular cooperativity, as confirmed by the decrease in the CRR size (Figure 2b).

**Table 1:** KWW Parameters Obtained for Pure CEL Glass and Its Mixtures with PVPVA

	τ_α_ (min)			β_KWW_			$\tau_{\alpha }^{\beta_{\mathrm{KWW}}}$		
Temperature (°C)	Pure CEL	Mixture with 2% PVPVA	Mixture with 5% PVPVA	Pure CEL	Mixture with 2% PVPVA	Mixture with 5% PVPVA	Pure CEL	Mixture with 2% PVPVA	Mixture with 5% PVPVA
30	27200	N.T.	N.T.	0.50	N.T.	N.T.	158	N.T.	N.T.
35	4870	2430	1890	0.48	0.67	0.87	58.9	186	709
40	647	981	1380	0.57	0.56	0.58	40.5	47.4	66.3
45	315	266	347	0.59	0.49	0.57	29.8	15.4	28.0

**Figure 4:**
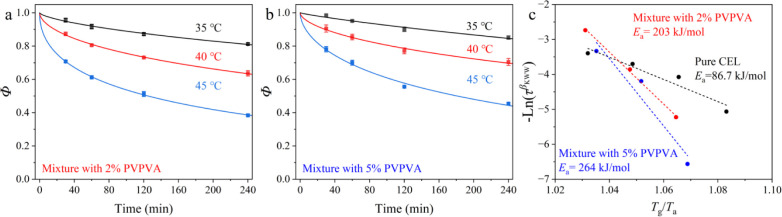
Fractions of the remaining excess enthalpy Φ of the mixtures with (a) 2% and (b) 5% PVPVA followed annealing at 35, 40, and 45 °C, fitted using the KWW function. (c) Arrhenius plots for the relaxation times of pure CEL and its mixtures with PVPVA. The data for pure CEL were adapted from ref 22.

Figure 5a presents an example of a dielectric loss spectrum obtained at 0 °C fitted with CC functions, where the three secondary relaxation processes (β, γ, and δ) were observed. Assignments of the secondary relaxations can be found in the literature.^32^ β*-*relaxation of pure CEL is ascribed to the Johari–Goldstein relaxation, that is, to a small-angle reorientational motion of the entire molecule affected by the glass transition (see below). The rotation of the phenyl ring with the sulfonamide group (Ph–SO_2_NH_2_) is represented by γ-relaxation, which is capable of hydrogen bond formation, and δ*-*relaxation is related to the rotation of the phenyl ring with the methyl group (Ph–CH_3_), which does not possess hydrogen bonding capability. Although the methylbenzyl ring does not have a dipole moment, the observed high intensity of the δ-relaxation stems from the fact that the rotations of the two phenyl rings are coupled. Specifically, when one ring rotates toward a perpendicular orientation relative to the other, it experiences repulsion from the highly polar Ph–SO_2_NH_2_ substituent. Consequently, the rotation of the low-polarity Ph–CH_3_ group induces significant fluctuations in the dipole moment due to this repulsive interaction with the polar sulfonamide ring during the rotational process.^33^

**Figure 5:**
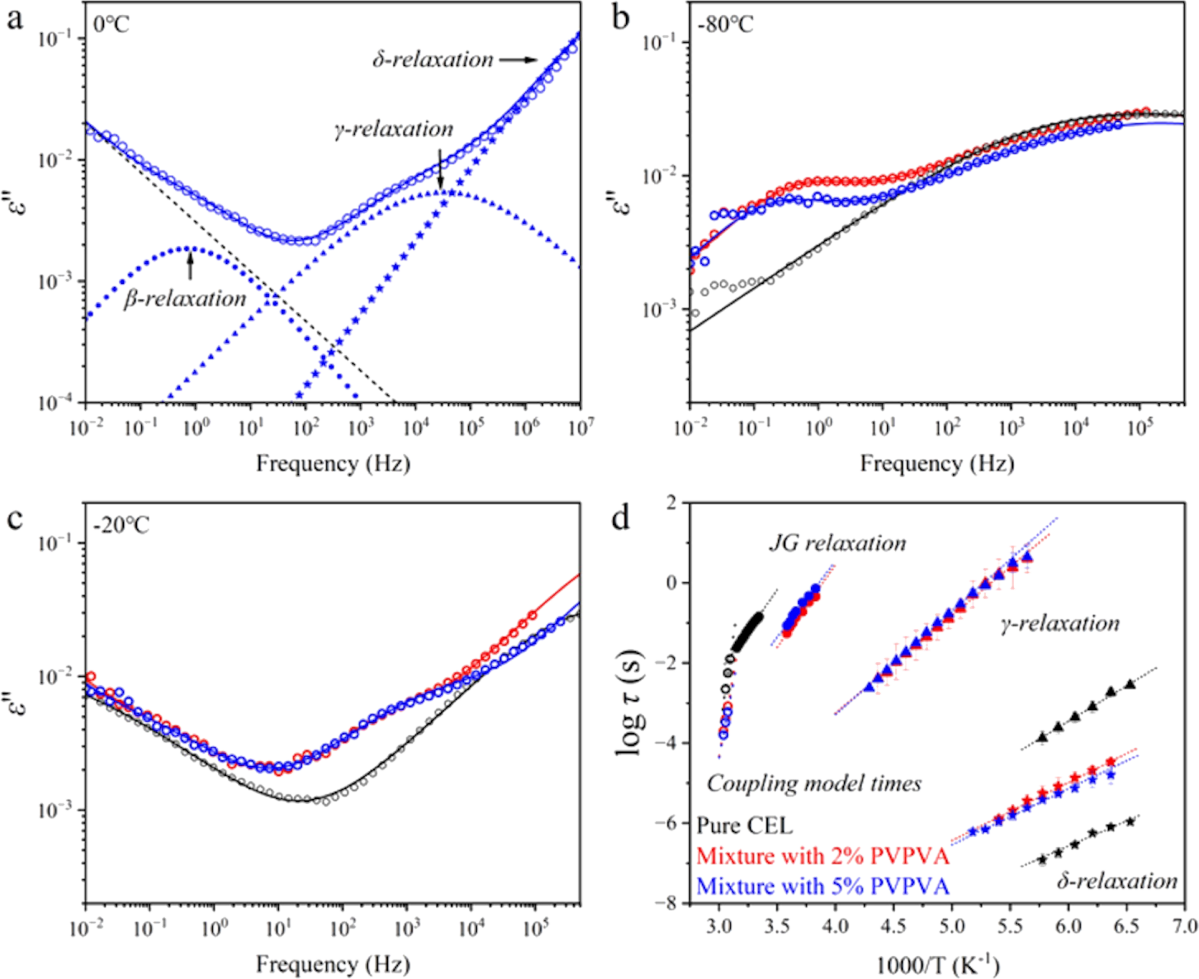
(a) Example of a dielectric loss spectrum fitted with three secondary relaxations for the mixture with 5% PVPVA obtained at 0 °C. Dielectric loss spectra of pure CEL glass and its mixtures with PVPVA obtained at (b) −80 and (c) −20 °C. (d) Secondary relaxation times as a function of reciprocal temperature fitted with the Arrhenius equation. Open circles show “precursor” relaxation times predicted by means of the Coupling Model.

The spectra obtained at different temperatures were similarly fitted. Figure 5b,c shows a comparison of the dielectric loss spectra of pure CEL glass and its mixtures with PVPVA at temperatures below *T*_g_. The spectra without deconvolution are also presented to demonstrate changes in the spectra free from possible artifacts originating from the deconvolution. The fitted results are presented in the Supporting Information. The strength of the γ-relaxation was enhanced and shifted to a lower frequency, indicating significantly restricted γ-mode local motions. Although this restriction may be attributed to the formation of hydrogen bonds between the sulfonamide group of CEL and the carbonyl groups of PVPVA, other origins including the appearance of an interfacial Maxwell–Wagner relaxation cannot be denied for explaining the drastic change in the shape of the spectra and the relaxation time. Similar observations were made for indomethacin glass mixed with PVPVA, where hydrogen bonds between indomethacin and PVPVA increased the secondary relaxation temperature range with increasing PVPVA loading.^34^ A shift to a higher frequency in the left wing was also observed in the BDS study for mixtures with PVPVA (Figure 5c), which is associated with a faster β*-*relaxation.

Figure 5d shows the secondary relaxation times as a function of the reciprocal temperature, where all of the secondary relaxation modes were significantly influenced. β-relaxation of the CEL glass appeared to be accelerated by adding PVPVA, whereas the mobility of γ*-* and δ-relaxations was suppressed. The activation energies for β*-*, γ*-*, and δ*-*relaxations of pure CEL glass were 79.1 ± 1.7, 33.9 ± 1.6, and 23.9 ± 2.4 kJ/mol, respectively. The acceleration of β-relaxation upon mixing with PVPVA did not accompany changes in the activation energy, which were 78.9 ± 2.1 and 72.8 ± 4.1 kJ/mol, respectively, for the mixtures with 2% and 5% PVPVA. However, adding 2% and 5% PVPVA increased the activation energy of γ-relaxation to 46.5 ± 0.9 and 49.4 ± 1.0 kJ/mol, respectively, in addition to prolonging the relaxation time. The activation energies for δ*-*relaxation slightly increased to 27.8 ± 0.5 and 26.8 ± 0.8 kJ/mol by adding 2% and 5% PVPVA, respectively. The significant changes in each relaxation time indicate that the origin of each mode may be different from the assignments made for the pure CEL glass. Nevertheless, even if it is the case, the disappearance of the original secondary relaxation times suggests a strong effect of PVPVA on the local molecular mobility of CEL glass.

The positive deviation of the α-relaxation peaks (Figure 3a) on the high-frequency side indicated the occurrence of Johari–Goldstein (JG) relaxation. According to the Coupling Model (CM), τ_JG_ should correspond well to the primitive relaxation time, τ_0_, of the CM equation, as follows:^35^

8 $$\tau_{JG}\approx \tau_{0}={\left(t_{c}\right)}^{n}{\left(\tau_{\alpha }\right)}^{1-n}$$

where *n* = 1 – β_KWW_. β_KWW_ was obtained by fitting the BDS spectra (Figure 3a), and 2 ps was used for *t*_c_.^36^

The τ_JG_ values of pure CEL glass and its mixtures are represented by the open circles in Figure 5d, where the τ_JG_ was fitted using the Arrhenius equation. The activation energies of pure CEL glass, the mixture with 2% PVPVA, and the mixture with 5% PVPVA were 381, 317, and 293 kJ/mol, respectively. For the mixtures with PVPVA, the JG relaxation was faster than that of pure CEL. JG relaxation is a precursor of α-relaxation and the local mobility responsible for crystallization in the glass state.^37^ One of the supposed mechanisms of diffusionless glass-to-crystal transformation, which is abnormally fast crystallization below *T*_g_, is the coalescence of homogeneous embryos. It is assumed to be controlled by JG relaxation.^38,39^ This JG relaxation is also likely the origin of the β-relaxation since they are consistent with relaxation times when *T* is close to *T*_g_. It can be observed that the temperature dependence of the β-relaxation and the calculated JG relaxation undergoes a change across *T*_g_, which has also been reported in previous studies.^35^

Figure 6 shows changes in the FTIR spectra of the CEL glass and PVPVA upon mixing. The stretching vibration bands of —C=O were observed at 1656 and 1730 cm^–1^ for pure PVPVA; however, they shifted to 1659 and 1736 cm^–1^ for the mixture with 2% PVPVA and to 1661 and 1735 cm^–1^ for the mixture with 5% PVPVA, respectively (Figure 6a). For the CEL glass, the absorption bands representing NH and SO_2_ stretching were observed at 3260 ^40^ and 1333 cm^–1^, respectively (Figure 6b). Notably, both bands shifted to higher wavenumbers with an increasing PVPVA content. Because these groups are involved in γ*-*relaxation, this observation supports their significant influence after adding PVPVA, as revealed by BDS measurements. The band for Ph–CH_3_ at 3030 cm^–1^ is related to δ-relaxation and was not influenced by PVPVA addition.

**Figure 6:**
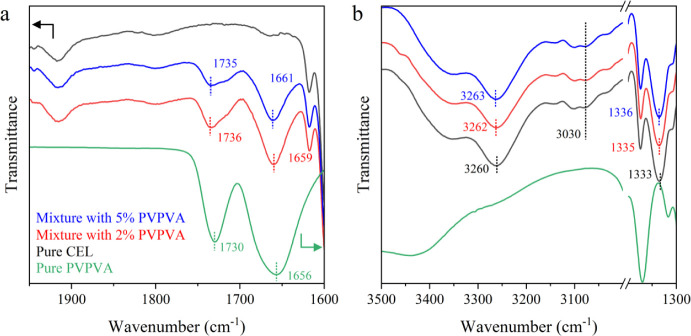
FTIR spectra of pure CEL, the mixture with 2% PVPVA, and the mixture with 5% PVPVA. (a) —C=O stretching vibration region at 1600–1900 cm^–1^. The scale for pure PVPVA was reduced to one-fifth. (b) −NH_2_ stretching vibration region at 3200–3500 cm^–1^, Ph–CH_3_ stretching vibration region at 3030 cm^–1^, and −SO_2_ stretching vibration region at 1300–1400 cm^–1^. For both figures, the spectra were vertically shifted for the sake of clarity.

### 3.4 Physical Stability of Pure CEL Glass and Its Mixtures with PVPVA

The physical stability of pure CEL glass and its mixtures with PVPVA was assessed at 60 °C and below *T*_g_ (35 and 50 °C). Figure 7 shows the DSC heating curves of the samples after storage. In all cases, *T*_onset_ decreased with an increasing storage time; however, the observation for pure CEL and mixtures seemed to have different origins. The crystallization of pure CEL glass was almost completed after 134 days at 50 °C, as observed from the disappearance of *T*_g_ in Figure 7b. Conversely, crystallization proceeded more slowly at 35 and 60 °C, requiring comparable storage periods to complete.^22^ In this temperature range, crystallization was likely proceeded based on the diffusionless glass-to-crystal transformation,^41^ where the maximum growth rate was 40–45 °C.^22,41^ Under all storage temperatures, the Δ*C*_p_ and crystallization enthalpy decreased owing to the decreased glass fraction, and the *T*_onset_ of cold crystallization decreased, indicating an increase in the crystal fraction in the stored samples in the case of the pure CEL glass (Figure 7a–c). On the other hand, an increase in crystallization enthalpy was observed for the mixtures with PVPVA without affecting Δ*C*_p_ (Figure 7d–i). The *T*_onset_ of cold crystallization decreased with time, similar to that observed for pure CEL glass. However, XRPD observations showed only a halo pattern for all mixtures with PVPVA after storage under all temperature conditions for up to 115 days, as shown in Supporting Information. Thus, the decrease in the *T*_onset_ was not because of an increase in the crystal fraction in the samples but due to an increase in the number of nuclei; i.e., the addition of PVPVA significantly decreased the nucleation rate of CEL glass and completely inhibited their growth. All samples were subjected to annealing at −20 °C to induce nucleation, which appeared successful for the pure CEL glass; however, it did not likely work well in the presence of PVPVA. The physical stability of the CEL glass was improved by adding only trace amounts of PVPVA despite a marginal increase in *T*_g_ (Figure 2b).

**Figure 7:**
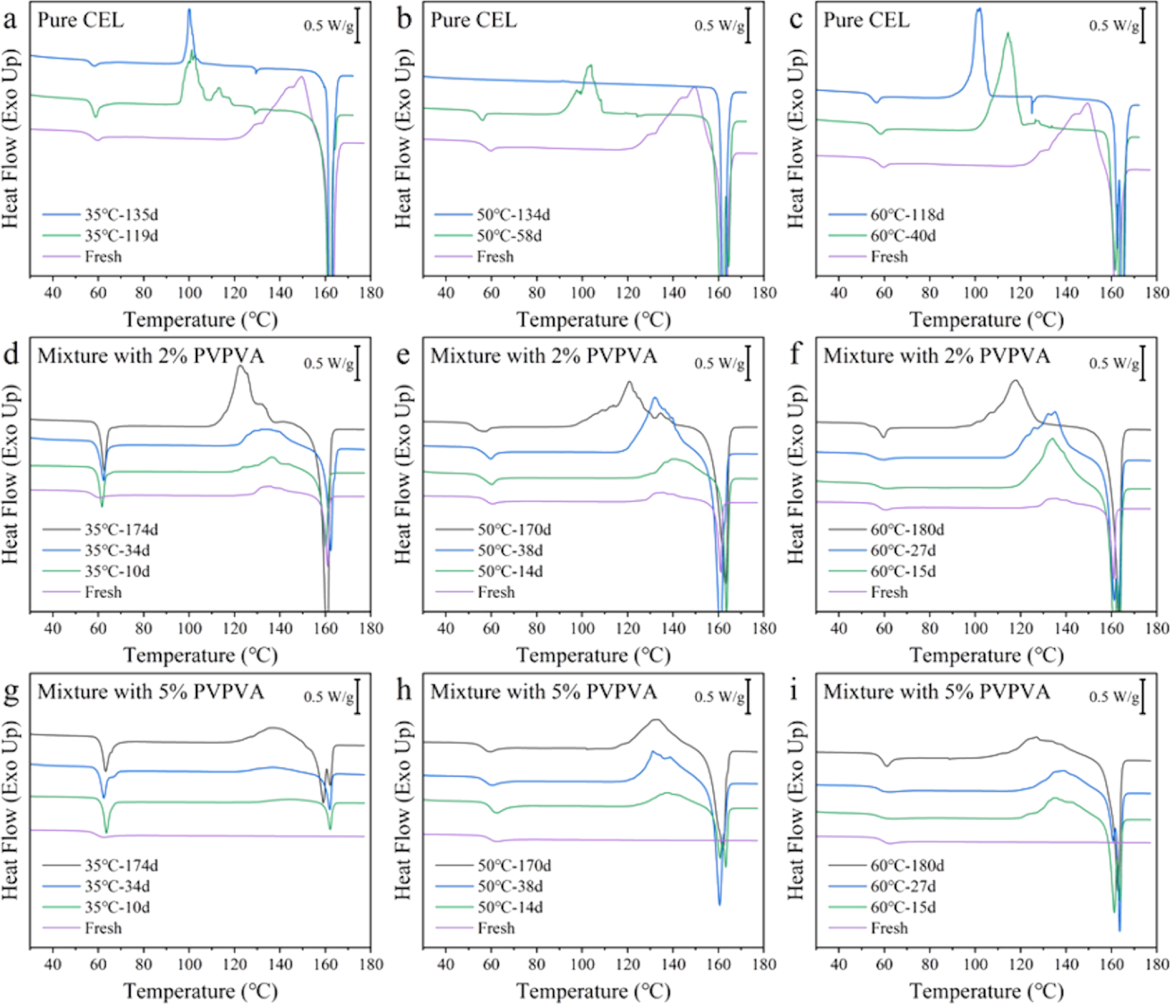
(a–i) DSC curves of pure CEL, the mixture with 2% PVPVA, and the mixture with 5% PVPVA following annealing at different *T*_a_. The storage temperature and periods are labeled.

Figure 8a–c presents the *T*_onset_ of cold crystallization. The enthalpy of cold crystallization of the mixtures with PVPVA is presented in Figure 8d–f. The enthalpy of cold crystallization tended to increase with storage time, except for the mixture with 2% PVPVA at 60 °C. The value for pure CEL glass is not presented, because it decreased with increasing crystallinity. The *T*_onset_ value of the mixture with 5% PVPVA was always higher than those of the mixture with 2% PVPVA and pure CEL, and the enthalpy was always smaller, suggesting that the initiation of crystallization was inhibited by a larger loading of PVPVA.

**Figure 8:**
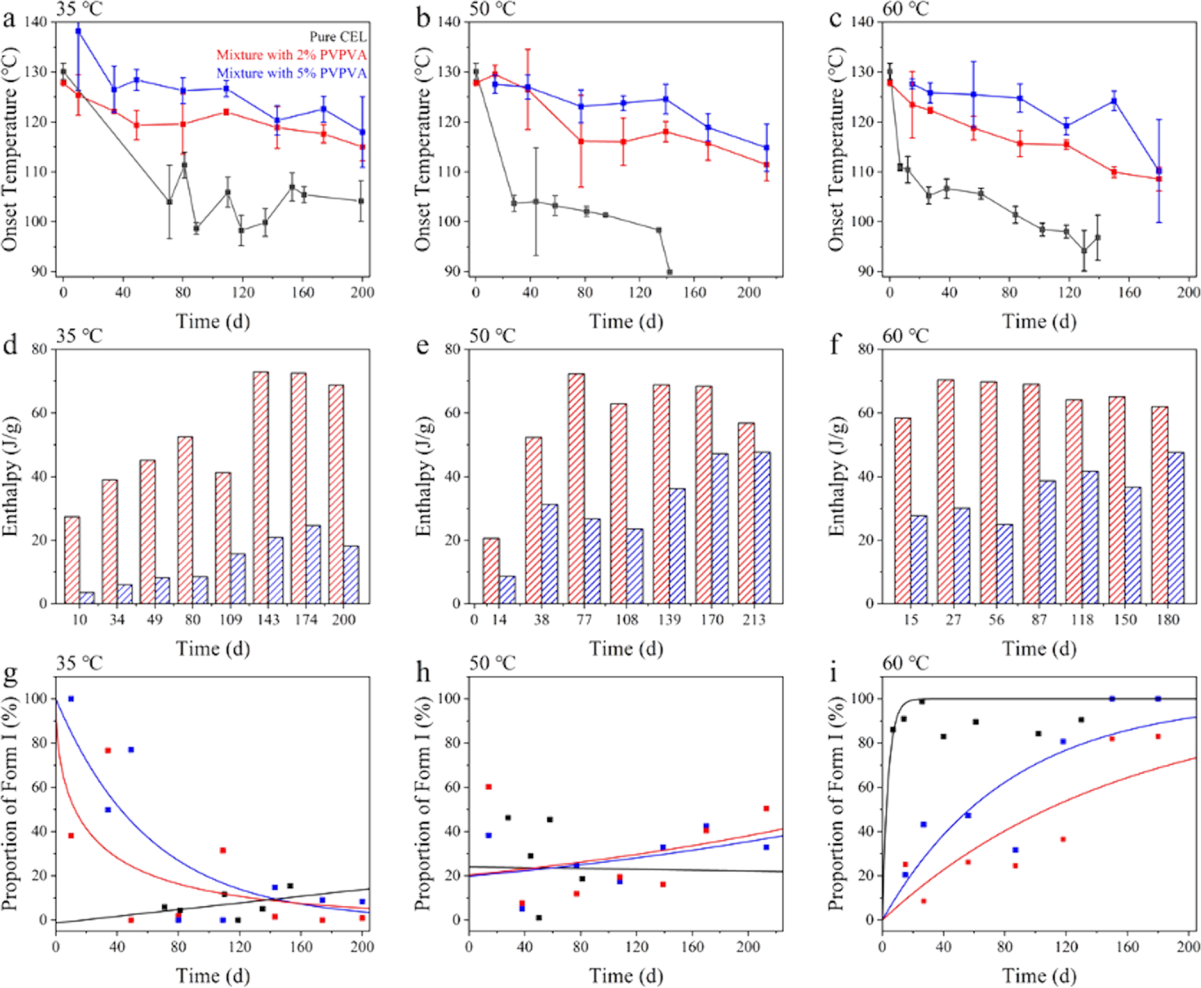
(a–c) Changes in *T*_onset_ of the cold crystallization peak for pure CEL, the mixture with 2% PVPVA, and the mixture with 5% PVPVA, represented by black, red, and blue, respectively, and stored at different temperatures. (d–f) Enthalpy of the cold crystallization peak of the mixtures with 2% and 5% PVPVA. That for pure CEL is not provided, as the values were influenced by the crystallinity of the stored samples as well as the number of nuclei. (g–i) Proportion of Form I after cold crystallization for pure CEL, the mixture with 2% PVPVA, and the mixture with 5% PVPVA. The storage temperature and periods are labeled.

In most cases, the melting behavior of pure CEL exhibited two peaks, where one belongs to Form III at 162 °C and the other is Form I at 164 °C. The proportions of the melting enthalpy of Form I are shown in Figure 8g–i. For pure CEL glass, the nuclei formed during the preparation process of the glassy state, which included annealing at −20 °C, are supposed to grow into Form III.^23^ It was observed during the storage at 35 °C; however, an increase in Form I was observed at 60 °C.^22^ In the presence of PVPVA, the resultant crystal was of Form I after short-term annealing at 35 °C, indicating insufficient nucleation at −20 °C. As nuclei of Form I can appear during DSC heating, it is therefore expected that Form I dominated in short-period annealing samples. The nuclei of Form III appeared to be formed slowly during the storage at 35 °C to increase the proportion of Form III after the cold crystallization. Because nucleation for Form I can proceed during annealing at 60 °C,^22^ an increased proportion of Form I with storage time was observed at 60 °C annealing. For the samples stored at 50 °C, an almost constant ratio of the two crystal forms indicated that nucleation of neither form was active at this temperature. The smaller cold crystallization enthalpies of the samples stored at 50 °C compared with those at 60 °C also supported this assumption. In summary, the presence of PVPVA likely suppressed nucleation to Form III at −20 °C as well as crystal growth during DSC heating.

Crystalline CEL can exist in five crystal forms (Form I, II, III, IV, and V).^40,42,43^ After annealing to induce the nuclei at −20 °C, pure CEL glass crystallized into Form III and I at 35 and 60 °C, respectively.^22^ Figure 9 shows the XRPD patterns of mixtures with PVPVA after storage. For both mixtures, the sample annealed at 60 °C presented intense diffraction peaks specific for Form I at 5.5°, 5.7°, 7.2°, and 16.6°. Peaks specific to Form III were also observed at 14.4°, 19.2°, and 21.1°. The crystallinity of the crystallized samples after storage at 50 °C was lower than expected from the DSC observations (Figures 7d–f and 8d–f). Most of the diffraction peaks were attributed to Form III and Form I. However, except for the characteristic peaks of Form I at 5.7°, 7.2°, and 16.6°, as well as Form III at 19.2°, those at 10.3°, 13.8°, and 17.7° indicated that Form II was more likely to be formed at 35 °C.

**Figure 9:**
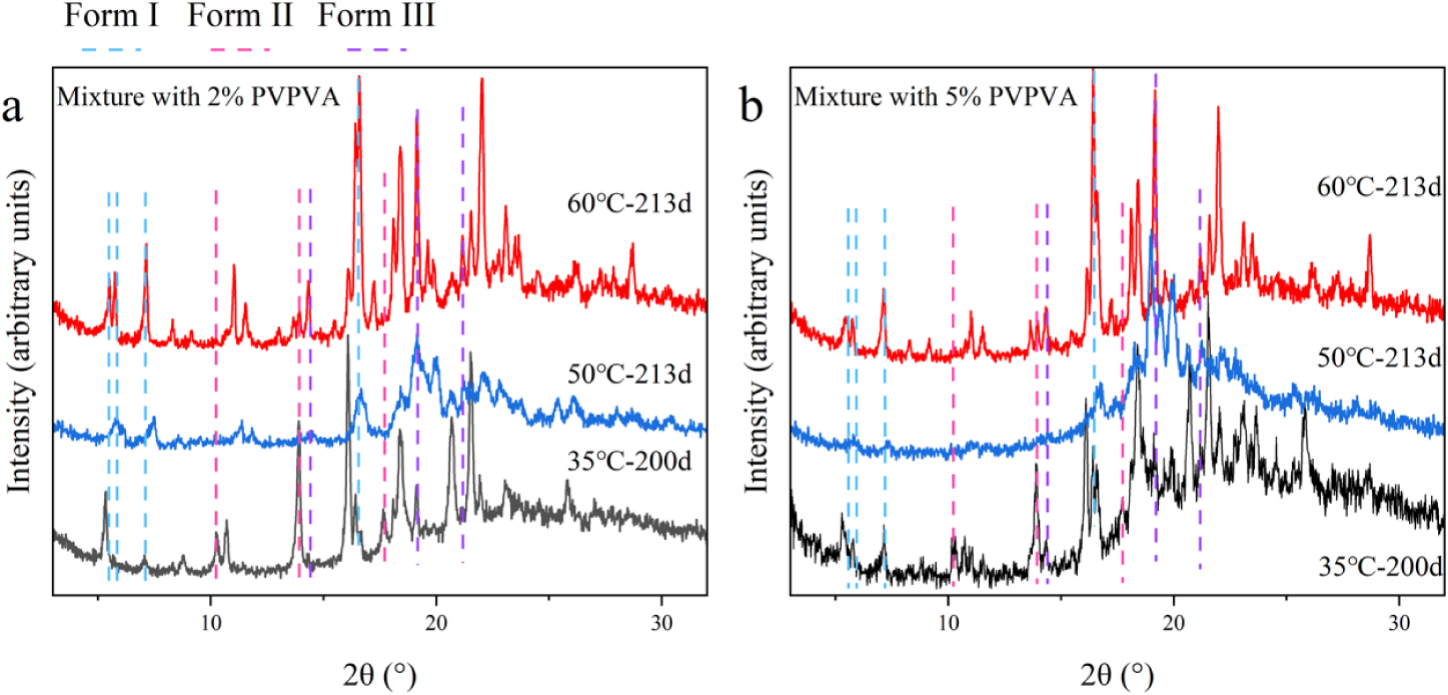
XPRD patterns for the mixtures with (a) 2% and (b) 5% PVPVA following the cold crystallization of the annealed samples at different temperatures. The storage temperature and periods are labeled.

### 3.5 Determination of the Nucleation Temperature of CEL in the Mixtures with PVPVA

The nucleation temperature is influenced by additives.^20,30^ One possible explanation is the change in molecular cooperativity.^30^ The effect of the addition of PVPVA on the nucleation behavior of the CEL glass was assessed by an annealing study using DSC (Table 2). The presence of nuclei increases the probability of cold crystallization and the crystallization enthalpy but decreases the *T*_onset_ of cold crystallization. Although the differences were marginal, the optimum temperature for nucleation in the presence of 2% or 5% PVPVA was approximately −60 °C. That for pure CEL glass for Form III was found at −50 °C in our previous study.^23^ Thus, the nucleation temperature decreased in the presence of trace amounts of PVPVA.

**Table 2:** Probability, Enthalpy, and *T*_onset_ of Cold Crystallization of CEL Glass in PVPVA Mixtures after Annealing at Various Temperatures for 5 min^a^

	Mixture with 2% PVPVA			Mixture with 5% PVPVA		
Annealing Temperature	Probability	Enthalpy (J/g)	*T*_onset_ (°C)	Probability	Enthalpy (J/g)	*T*_onset_ (°C)
–70 °C	1.0	40.6 ± 18.6	129.4 ± 4.4	0.9	2.2 ± 2.3	131.9 ± 9.8
–60 °C	1.0	51.4 ± 19.9	128.3 ± 1.6	1.0	6.5 ± 5.5	131.8 ± 2.1
–50 °C	0.9	49.3 ± 21.8	128.5 ± 3.2	0.9	4.1 ± 6.8	133.1 ± 1.8
–40 °C	0.6	51.7 ± 24.9	128.3 ± 2.2	0.8	5.8 ± 11.1	130.4 ± 2.9

a *n* = 10.

In this study, the physical stability was assessed after annealing at −20 °C. The probability of cold crystallization after annealing at −20 °C for 1 h was 100% for pure CEL glass.^23^ However, it decreased to 50% and 40% in the presence of 2% and 5% PVPVA, respectively (Table S1), owing to the shift in the nucleation temperature to a lower temperature. The DSC results suggested the formation of nuclei during the storage even for the mixtures with PVPVA, as cold crystallization was observed for the samples after long-term storage (Figure 7d–i). However, the number of nuclei should be much smaller than pure CEL, and this is likely the key factor for the stabilization. Additional factors for the stabilization are provided below.

### 3.6 Growth of CEL Crystals under PLM

Figure 10 shows the PLM image of CEL crystal growth from the mixture with 2% PVPVA and an analysis of the growth rate at 120 °C. The mean growth rates for pure CEL and the mixture with 2% PVPVA were 3.08 ± 1.65 and 4.61 ± 2.36 μm/s, respectively. Thus, no statistically significant difference in the overall growth rate was observed with the addition of PVPVA. However, analysis of the growth rate distribution revealed that pure CEL glass was centered at approximately 2 μm/s, whereas the mixture with PVPVA was split into two peaks at approximately 1 and 5 μm/s. Therefore, the growth rate was partially suppressed by the adsorption of PVPVA on the growth surface, which allowed acceleration of the growth of the nonadsorbed surface. Distortion of the crystal shape and ignorable impact on the growth rate by the addition of a small amount of polymer agrees with our previous observation for ibuprofen glass.^30^

**Figure 10:**
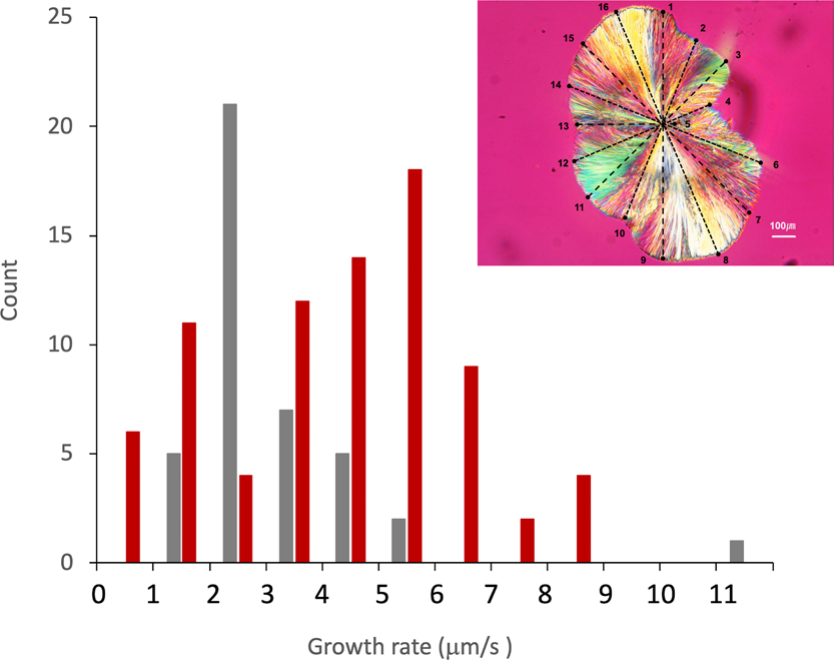
Distribution of CEL crystal growth rates with (red) or without (gray) 2% PVPVA. The inset shows an example of the analysis for CEL/PVPVA mixtures, where 16 lines from the center of the crystal are presented. The Experimental Section presents further details of the analysis.

## 4 Discussion

### 4.1 Roles of Molecular Mobility and Molecular Interactions in Inhibiting Crystallization

A much larger amount of polymer relative to the drug has generally been used to stabilize the glassy state of commercial ASDs, where several stabilization mechanisms are expected to be at work, including direct molecular interactions between the polymer and drug to decrease the molecular mobility of the drug, steric hindrance by the polymer to decrease the interaction frequency of the drug molecules, and increasing the viscosity to slow molecular diffusion. However, even trace amounts of the polymer can exhibit a strong stabilization effect against crystallization. BDS and DSC measurements revealed that α*-*relaxation was slowed down with increasing amounts of PVPVA both above and below *T*_g_. This could be explained by the formation of hydrogen bonds between the CEL and PVPVA molecules. The increase in the viscosity in the presence of PVPVA may be partially responsible for the observed decrease in the molecular mobility. As global molecular mobility is frequently shown to negatively correlate with physical stability,^1,8,44,45^ even the addition of trace amounts of PVPVA is expected to have a positive impact on the physical stability of CEL glass. Similar stabilization effects with small amounts of polymer have been previously reported. For example, prepared by combining solvent evaporation and melt-quenching, ketoconazole glass crystallized within 3 days at 40 °C; however, the crystallization was not observed for the mixture with 4% poly­(acrylic acid) for more than 1 year.^46^ Moreover, indomethacin glass prepared using solvent evaporation crystallized within 2 weeks at 30 °C; however, no crystallization was observed for a mixture with 5% PVPVA over 20 weeks.^47^ Furthermore, the α*-*relaxation time of nifedipine glass decreased with an increasing amount of PVP.^48^ The onset time of isothermal crystallization exhibited a correlation with the relaxation time in the PVP concentration range of 2.5–10%, indicating the importance of molecular mobility for crystallization.

JG relaxation is also generally expected to become slower with the addition of polymers. For example, a slower JG relaxation of an indomethacin–PVPVA mixture was observed when the load of PVPVA increased from 40% to 95%.^34^ That for CEL glass was suppressed by adding 10% of octaacetylmaltose, whereas no significant impact on the α*-*relaxation was observed upon its addition.^32^ However, as local mobility does not depend on the molecular weight of the entire molecule, the opposite effect can be observed by adding polymeric excipients, as well. The addition of 1% poly­(ethylene oxide), polybutadiene, and polyisoprene enhanced the crystallization of nifedipine, which was explained by the enhancement of local molecular mobility.^49,50^ In our observations, the physical stability of the CEL glass was enhanced despite the speedup of JG/β-relaxation. Thus, these modes of molecular mobility have less impact on the physical stability of CEL glass.

The mobility of γ*-* and δ*-*relaxation of the CEL glass was significantly suppressed by the addition of PVPVA. Although various interpretations are possible for explaining the drastic change in these relaxation times, the effect on γ*-*relaxation may be explained by the formation of hydrogen bonds between CEL and PVPVA. As γ- and δ-relaxations are likely to be coupled, it also explains the effect on the δ*-*relaxation. Similar observations in the literature include the acceleration of the γ*-*relaxation of indomethacin glass in the presence of 3% poly­(ethylene oxide).^51^ In a study by Grzybowska et al.,^33^ the addition of 10% octaacetylmaltose suppressed the strength of γ*-*relaxation of CEL glass. This observation indicated that the mobility of the sulfonamide group was suppressed via hydrogen bond formation with the carbonyl group of octaacetylmaltose. Also presented in their study was the ineffectiveness of the carbonyl group of PVP to slow down γ*-*relaxation,^32^ although it also forms hydrogen bonds with CEL. Consistent with their results, our study indicated the deceleration of the γ*-*relaxation of CEL glass by interacting with PVPVA. Thus, the vinyl acetate unit may be involved in the suppression of γ*-*relaxation mobility. Hydrogen bonding between the sulfonamide group and nitrogen of the pyrazole ring is responsible for the formation of CEL crystals.^52^ Because the mobility of the former group is related to γ*-*relaxation, its activity and availability should significantly influence the crystallization behavior. Thus, it is reasonable to assume that the suppression of the γ*-*relaxation mobility inhibits the nucleation of the CEL glass. The stabilization mechanism of trace amounts of PVPVA should be the same, at least partially, with that observed for octaacetylmaltose.^32^

### 4.2 Role of Molecular Cooperativity in Inhibiting Crystallization

The stabilization mechanism of CEL glass in the presence of PVPVA likely includes molecular interactions that suppress the α, γ, and δ modes of molecular mobility. However, it cannot be assumed that only a small percentage of additives suppresses the mobility of all drug molecules via direct molecular interactions. Thus, additional mechanisms for stabilizing the glassy state are needed for an explanation.

The nucleation temperature is generally found slightly above *T*_g_.^26,53^ However, in some cases, the nucleation temperature has been reported at temperatures much lower than *T*_g_.^22,23,54,55^ For CEL glass, the nucleation temperature for Form III was in the freezing temperature range, which was lower than *T*_g_ by more than 100 °C. The enhanced crystallization of glass that experiences freezing temperatures is sometimes explained by mechanical activation due to crack formation;^56^ however, it was not likely for CEL glass because the optimum temperature for nucleation was found at −50 °C.^23^ The annealing at temperatures lower than −50 °C, where cracks were also formed, was less effective. Due to the severely restricted molecular mobility below *T*_g_, the nucleation mechanism there may be distinct from that prevailing above *T*_g_. Feature of compounds that can exhibit nucleation at low temperatures is presumably capable of forming nuclei only with local molecular mobility without molecular rearrangement. Thus, inhibition of the moieties that form hydrogen bonds is one of the key factors in suppressing nucleation, as discussed in the previous section.

Moreover, additives influence the nucleation temperature, which was observed in the CEL/PVPVA mixtures (Table 2). This observation indicates the influence of the additive on temperature-dependent dynamic processes. The decreased CRR size of the CEL glass after the addition of PVPVA indicates a decrease in molecular cooperativity (Figure 2b). In our study, nucleation was induced by annealing at −20 °C. Owing to the decrease in nucleation temperature in the presence of PVPVA, the probability of crystallization after annealing at −20 °C decreased (Table S1), which was attributed to changes in molecular cooperativity. Thus, the stabilization effect of trace amounts of additive, which may have difficulty affecting the dynamics of the entire glassy molecule via direct interaction, can be explained by its influence on the dynamic properties of the entire system by changing the molecular cooperativity.

### 4.3 Influence of Trace Amounts of PVPVA on CEL Crystal Growth

The *T*_onset_ and enthalpy of the cold crystallization of pure CEL glass after annealing at −20 °C were approximately 117 °C and 72 J/g, respectively.^23^ A significant decrease in the crystallization enthalpy and an increase in the crystallization temperature were observed in the presence of trace amounts of PVPVA (Tables 2 and S1). This observation indicates that the crystal growth process and nucleation might be significantly influenced by trace amounts of PVPVA.

In addition to the decrease in molecular mobility, another explanation for the suppression of crystal growth is the poisoning of the growth surface of crystals.^57–59^ PLM observations confirmed that the presence of PVPVA influenced the growth rate distribution owing to the partial adsorption of PVPVA onto the interface; however, the overall crystallization rate was not affected. Therefore, although surface poisoning was likely to occur, its effect on the growth rate was negligible. The increase in *T*_onset_ of cold crystallization and the decrease in enthalpy should primarily align with the decrease in the number of nuclei.^60^ In summary, the effect of trace amounts of PVPVA was primarily due to the inhibition of nucleation rather than the suppression of crystal growth.

## 5 Conclusion

The molecular dynamics and physical stability of CEL glass and its mixtures with trace amounts of PVPVA were investigated using BDS and DSC. The isothermal crystallization studies at 35–60 °C revealed that adding trace amounts of PVPVA significantly improved the physical stability of CEL glass. The α*-*relaxation of CEL glass was suppressed both above and below *T*_g_ in the presence of PVPVA, whereas JG relaxation was accelerated. The suppression of α-relaxation likely originated from hydrogen bonding between CEL and PVPVA. However, this was not sufficient to explain the strong stabilization effect. Adding PVPVA significantly suppressed the mobility of γ*-* and δ*-*relaxation of the CEL glass. In particular, the effect on γ*-*relaxation likely played an important role, because the sulfonamide group, which is involved in γ*-*relaxation, needs to form hydrogen bonds with another CEL molecule for crystallization. Moreover, the nucleation temperature was lowered by adding PVPVA, thus decreasing the probability of crystallization. The effect of trace amounts of PVPVA was primarily due to the inhibition of nucleation rather than the suppression of the crystal growth process. This study provides important information for the stabilization of ASDs using polymeric excipients.

## Supplementary Material



## Notes

Dielectric loss spectra of CEL glass and its mixtures with PVPVA, methodology of masterplot, XPRD patterns for CEL and its mixtures with PVPVA after annealing at 50 °C for 115 days, probability, enthalpy, and onset temperature of cold crystallization of CEL/PVPVA mixtures after annealing at various temperatures for 1 h, and detailed fitted results of Figure 5 (PDF)
