# Supplementary material for: Influence of the Addition of Trace Amounts of Vinylpyrrolidone–Vinyl Acetate Copolymer (PVPVA) on the Crystallization of Celecoxib Glass
Source: Mol Pharm. 2025 Dec 15;23(1):280–92. doi: 10.1021/acs.molpharmaceut.5c00934 (PMC12776578; doi:10.1021/acs.molpharmaceut.5c00934)
Supplement: Supplementary file 1 [file mp5c00934_si_001.pdf]

Supporting information

Influence of Addition of Trace Amount of Vinylpyrrolidone-Vinyl Acetate Copolymer (PVPVA) on Crystallization of Celecoxib Glass

Xue Han[1,2], Kaoru Ohyama[1], Kohsaku Kawakami[1,2,*]

[1] Research Center for Macromolecules and Biomaterials, National Institute for Materials Science, 1-1 Namiki, Tsukuba, Ibaraki 305-0044, Japan

[2] Graduate School of Science and Technology, University of Tsukuba, 1-1-1 Tennodai, Tsukuba, Ibaraki 305-8577, Japan

* Corresponding author

E-mail: kawakami.kohsaku@nims.go.jp, Tel. +81-29-860-4424

1. The dielectric loss spectra in Figure S1 shows that the peak of α relaxation moved to higher frequencies with the increasing temperatures when $T > T_g$. The strength of α relaxation peak ($\Delta\varepsilon$) is directly related to the quantity of units, at the crystallization onset temperature (T_c), a significant decrease in the magnitude of $\Delta\varepsilon$ signifies the initiation of recrystallization within the sample. The T_c for pure CEL, mixtures with 2% PVPVA and mixtures with 5% PVPVA were 94, 100, and 106 °C, respectively.

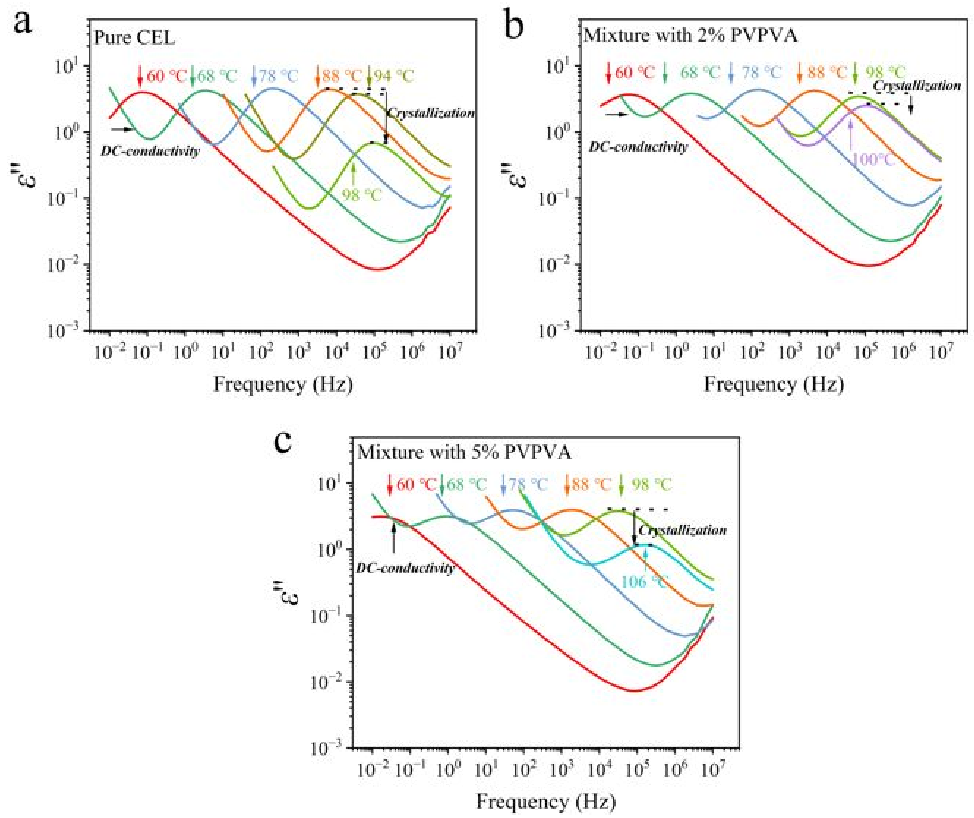

Figure S1 The dielectric loss spectra of (a) pure CEL, (b) mixtures with 2% PVPVA and (c) mixtures with 5% PVPVA when temperatures over T_g. The black dashed lines represent the beginning of recrystallization accompanied by a decrease in $\Delta\varepsilon$.

The methodology for confirming τ_α values used for calculation of τ_{JG} is masterplot, which is validated by the distinct dielectric loss peak observed at higher temperature, which shifts systematically to lower frequencies upon cooling. Take pure CEL as an example, the full peak profile at 58 °C serves as a reference for reconstructing partially resolved peaks at lower temperature (50 °C). Firstly, the τ_α of pure CEL at 58 °C is confirmed, then horizontally shifting the spectra to achieve superposition with the master curve, we compensate for incomplete peak resolution in the glassy state. The frequency of τ_α shifts to the lower side, and according to the equation: $\tau_\alpha = 1/(2\pi f)$, the τ_α of pure CEL at 50 °C can be speculated.

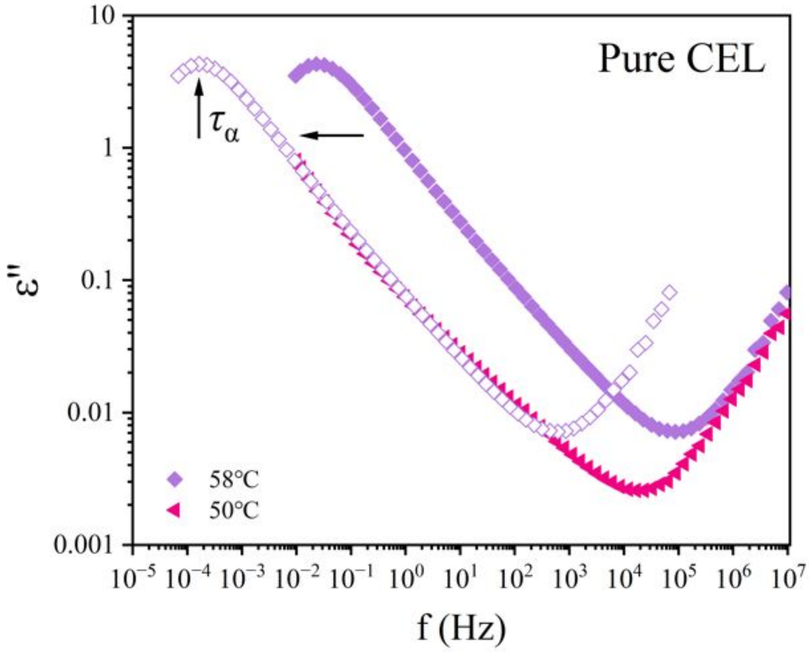

Figure S2 The dielectric loss spectra of pure CEL at 50 and 58 °C.

2. The annealed samples at 50 °C were collected and subjected to an XRPD measurement. For mixtures, no crystallization characteristic peaks were found in the pattern. The pure CEL presented diffraction peaks for Form I at 5.5°, 16.6° and for Form III at 14.0°, 16.2°, 21.6°.

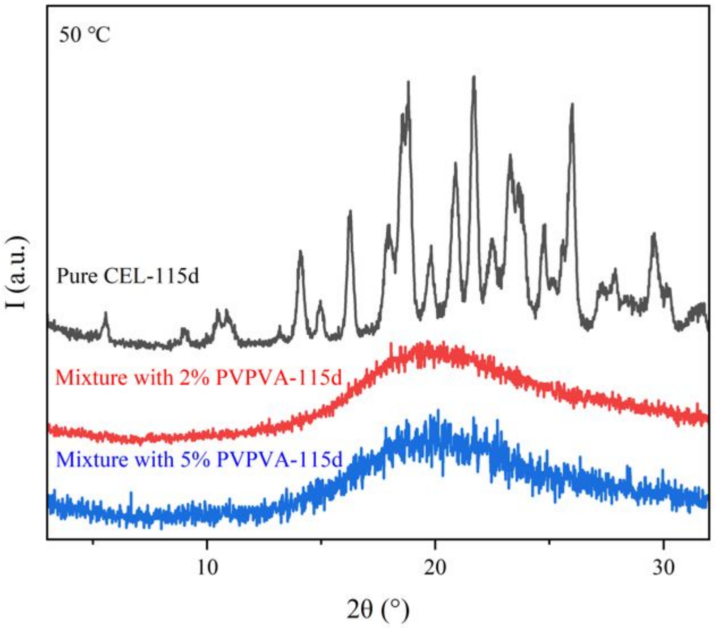

Figure S3 XPRD patterns for pure CEL, mixture with 2% PVPVA and mixture with 5% PVPVA after annealing at 50 °C for 115d.

3. Table S1 The probability, enthalpy and onset temperature (T_{onset}) of cold crystallization of mixtures after annealing at various temperatures for 1 h.

Annealing Temperature	Mixture with 2% PVPVA			Mixture with 5% PVPVA		
	Probability	Enthalpy (J/g)	T_{onset} (°C)	Probability	Enthalpy (J/g)	T_{onset} (°C)
-80 °C	0.9	45.6±27.8	127.4±2.3	0.9	5.8±8.5	130.7±3.0
-60 °C	0.8	40.8±19.5	128.2±2.8	0.8	9.3±8.7	132.7±3.2
-40 °C	0.8	42.2±25.0	128.2±1.5	0.8	6.3±7.9	132.6±2.2
-20 °C	0.5	42.5±29.0	130.3±3.6	0.4	2.3±2.5	134.4±6.4
0 °C	0.33	21.9	133.2	0.67	4.8	131.6

n=10 except for 0°C (n=3)

4. Figure S4 shows the detailed deconvoluted results of Figure 5.

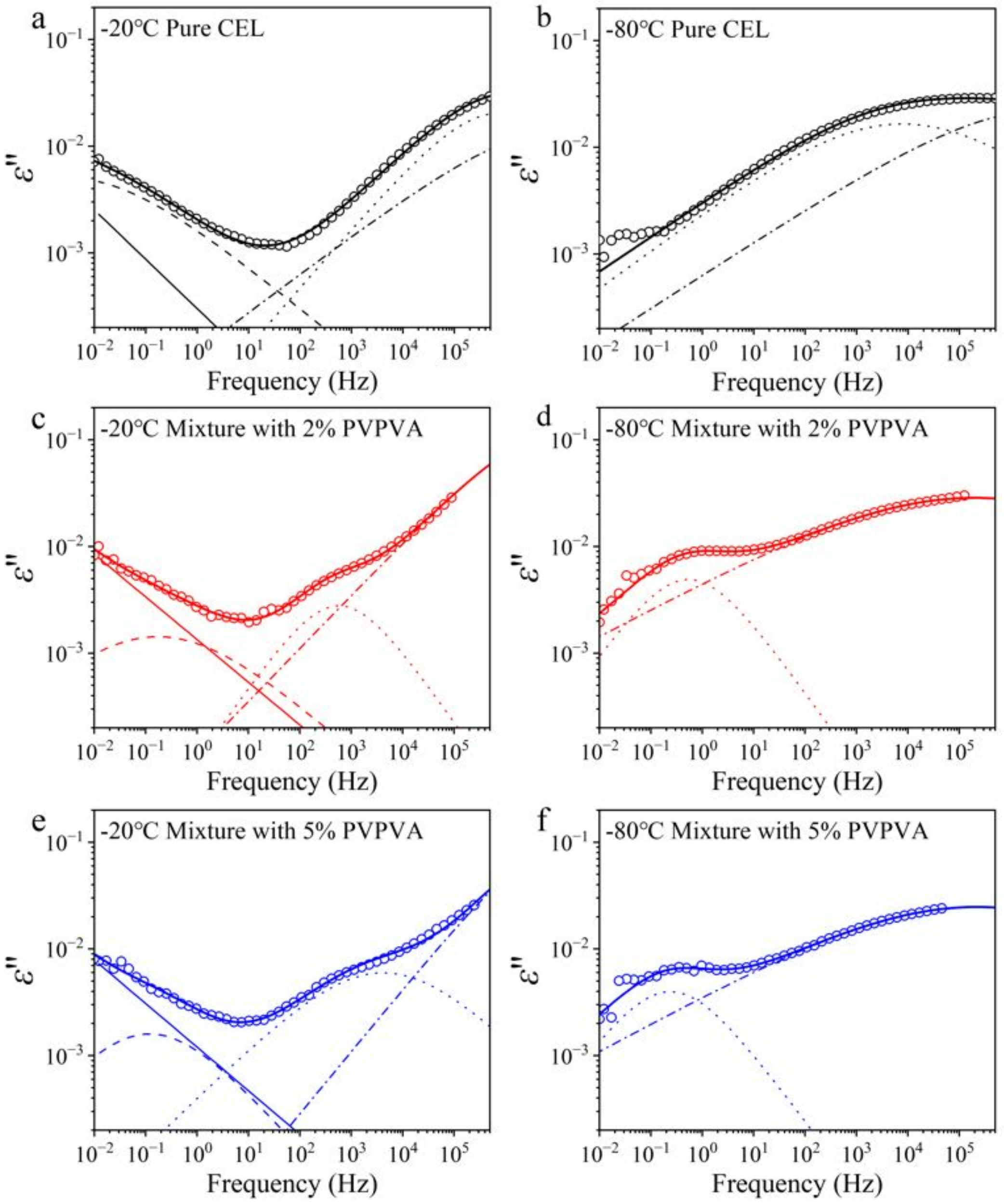

Figure S4 The deconvoluted results for pure CEL, mixture with 2% PVPVA and mixture with 5% PVPVA obtained at -20 °C and -80 °C, which was deconvoluted into three secondary relaxations. The order from low frequency to high frequency is the wing of α relaxation, β relaxation, γ relaxation, and δ relaxation at -20 °C; only γ relaxation, and δ relaxation appeared at -80 °C.
